# Data Transmission Efficiency in Bluetooth Low Energy Versions

**DOI:** 10.3390/s19173746

**Published:** 2019-08-29

**Authors:** Patricio Bulić, Gašper Kojek, Anton Biasizzo

**Affiliations:** 1 Faculty of Computer and Information Science, University of Ljubljana, 1000 Ljubljana, Slovenia; 2 Computer Systems Department, Jozef Stefan Institute, 1000 Ljubljana, Slovenia

**Keywords:** Bluetooth Low Energy (BLE), power estimation, performance evaluation, Internet of Things (IoT), Wireless Sensor Network (WSN)

## Abstract

One important aspect when choosing a Bluetooth Low Energy (BLE) solution is to analyze its energy consumption for various connection parameters and desired throughput to build an optimal low-power Internet-of-Things (IoT) application and to extend the battery life. In this paper, energy consumption and data throughput for various BLE versions are studied. We have tested the effect of connection interval on the throughput and compared power efficiency relating to throughput for various BLE versions and different transactions. The presented results reveal that shorter connection intervals increase throughput for *read/write* transactions, but that is not the case for the *notify* and *read/write without response* transactions. Furthermore, for each BLE version, the energy consumption is mainly dependable on the data volume. The obtained results provide a design guideline for implementing an optimal BLE IoT application.

## 1. Introduction

In recent years we have seen a significant progress in digital technologies, which contributes to the ever-present concept of Internet of Things (IoT). IoT has become a very important industrial segment. It involves connected smart devices that process and share all types of data between each other and can be controlled via the Internet. As a result, several low-power hardware platforms have become available to build IoT solutions [1]. In this context, energy-efficient short-range wireless communication technologies have become a hot topic for research and development [2]. Among available solutions, Bluetooth Low Energy (BLE) is gaining more and more popularity [3]. Small, embedded sensors that communicate via BLE are used in everyday life for a wide number of applications such as wearable devices, smart homes, home automation, e-health systems, indoor positioning, traffic control, and telemedicine [4,5,6,7,8,9,10,11,12,13,14,15,16,17,18,19,20,21,21].

BLE is a short-range wireless communication technology, developed by the Bluetooth Special Interest Group (SIG), aiming at low-cost and low-power short-range communication [22,23]. BLE has very low power consumption, and devices that use BLE for communication can be powered by coin-cell batteries and can operate for months [24].

The BLE protocol stack is composed of two main parts: the controller and the host. The controller comprises the physical layer and the link layer and is typically implemented as a small System-on-Chip (SOC) with integrated radio. The host runs on an application processor and includes upper layer functionality [2]. An in-depth description of the BLE protocol, highlighting the main characteristics and implementation details can be found in Reference [3]. Bluetooth 5 has been the most significant advancement in the Bluetooth versions since the introduction of BLE in Bluetooth 4.0. Bluetooth 5 significantly increases the range, speed, and broadcast messaging capacity of Bluetooth applications and makes many previously unfeasible use cases in smart home automation, enterprise, and industrial markets a reality. The technical paper of Reference [23] provides an in-depth look at the Bluetooth 5 core specification. In short, there are four significant new features of Bluetooth 5: a higher bit rate of 2 Mbps; a long-range mode with better sensitivity at two new lower bit rates of 500 kbps and 125 kbps; an eight-times improvement in broadcast capability with advertising extensions; and an improved channel selection algorithm.

When choosing an embedded platform to build an optimal low-power application and extend battery life, one fundamental aspect is to analyze the power consumption of its components. This is also true for BLE. To address this challenge, this paper compares energy consumption relating to throughput for different connection parameters and various BLE versions. It aims to identify the connection parameters needed to achieve optimal power efficiency in BLE communication.

The paper is organized as follows. Section 2 reviews the related work. Section 3 describes the hardware used in the measuring system, test scenarios, and measurement methods. Section 4 presents and discusses the results. Finally, Section 5 concludes the paper.

## 2. Related Work

To the best of our knowledge, there is no study which would compare data transmission efficiency for various versions of BLE. Such a comparison would be even more interesting with the arrival of Bluetooth 5 [23], which introduces many innovations that are incompatible with previous versions without modifying the physical layer of the device.

Garcia-Espinosa et al. [1] presented the energy consumption of four BLE 4.2 commercial platforms (Arduino 101 (Intel A-101), Texas Instruments (TI) CC2540, NXP FRDM-KW41Z, and Cypress Semiconductor CY8CKIT-042-BLE-A) focusing on the consumption of the system when the platform is being configured as a BLE central device and as a peripheral device. Additionally, a guide for IoT designers is provided to calculate the battery life for several connection intervals to help designers deliver much more dependable low-energy IoT systems. Furthermore, the authors pointed out that measuring the energy consumption in applications that demand different data throughput is required to provide a full design guideline for implementing an optimal BLE IoT application. Tosi et al. [3] provided an in-depth description of the BLE protocol, highlighting the main characteristics and implementation details, and reviewed the state of the art on BLE characteristics and performance. In particular, the authors analyze throughput, the maximum number of connectable sensors, power consumption, latency, and maximum reachable range to identify what are the current limits of BLE technology. The work is limited to BLE 4.2 only. Cho et al. [24] created an accurate analytical model to describe selected BLE 4.0 properties such as the discovery latency, as well as the discovery probability. The model was also validated via extensive simulation experiments. The authors found out that the inappropriate parameter settings considerably impair the efficiency of BLE 4.0 devices. A comparison of energy consumption between BLE 4.0 and ZigBee/802.15.4 can be found in Reference [25]. In addition to energy consumption, the authors tested the effect of interference on BLE and ZigBee. Another study comparing BLE with other wireless technologies in regards to energy consumption was made in Reference [26]. The results revealed that BLE 4.0 provides an inexpensive and power-efficient solution for wireless communication. Hortelano et al. [27] evaluated BLE and proposed it as the ideal candidate where total coverage, zero fails, scalability, and sustainability are important due to its low power consumption. This study is limited to BLE 4.0/4.1 only. An evaluation of the BLE discovery procedure based on new features in Bluetooth 5.0 (i.e., an adapted version of scannable undirected advertising events) has been presented in Reference [28]. The theoretical maximum data throughput of different BLE versions was studied in Reference [29]. Masouros et al. [30] explored a three-layered architecture able to acquire, process, and store healthcare data as well as to provide real-time ECG signal arrhythmia detection. The performance of the classic Bluetooth protocol and BLE 4.1 was quantified in terms of packet transmission delay, CPU utilization, and power consumption. The packet delay between two successive received packets was measured. The experiments tried to capture the variation in the transmission delay of the protocol in a real-life scenario with increasing distance between transmitter and receiver as well as with the inclusion of obstacles like walls. Moreover, all experiments were conducted in a non-isolated environment with Wi-Fi networks present and the interference is included in the measured delay. The results showed that the packet loss in BLE 4.1 significantly increases when the distance between transmitter and receiver is larger than six meters and with three obstacles in the transmission path.

## 3. Materials and Methods

### 3.1. Hardware

To evaluate the energy consumption of BLE, we have used two Nordic *nRF52840 DK* [31] development kit boards (Nordic Semiconductor, Trondheim, Oslo, Norway ), one as the central device and the other as the peripheral device. The *nRF52840 DK* is a single-board development kit for Bluetooth development on *nRF52840 SoC* [32] (Nordic Semiconductor, Trondheim, Oslo, Norway), which is designed around an ARM Cortex-M4 CPU (Arm Holdings, Cambridge, England, UK). It has a dynamic multi-protocol transceiver that supports various Bluetooth protocols. It has the full hardware and software support for all the new Bluetooth 5 features: long range (coded) and high-speed PHY layers, advertising extensions, and improved coexistence with other wireless devices. The Nordic protocol stacks are known as *SoftDevices* [33] (Nordic Semiconductor, Trondheim, Oslo, Norway ). The *nRF52840 SoC* is supported by the S140 SoftDevice, which is BLE control and peripheral protocol stack that supports an advertiser and an observer role and is capable of running up to twenty concurrent connections.

The central device is the measurement initiator and controller and is connected to a personal computer for test monitoring. The peripheral device is the device under test, which is configured to support only BLE operations, and it is decoupled from on-board peripheral devices such as LEDs, interface MCU, and external memory. BLE is backwards compatible down to version 4.0, which is why a single chip (*nRF52840 SoC*) was used for all tests. Tests for versions BLE 4.0/4.1 and BLE 4.2 were performed with the same dynamic multi-protocol transceiver as tests for BLE 5, with parameters set to that specific version capabilities, as described in Section 3.4.

### 3.2. Measuring System

To measure the energy consumption, *Power Profiler Kit* [34] from Nordic Semiconductor (Trondheim, Oslo, Norway) was used. It is a current measurement tool for embedded development, specifically optimised for measuring the power consumption of Bluetooth devices. *Power Profiler Kit* has a three-stage analog measurement circuit, which splits the total current range into three ranges: 1 μA–70 μA, 70 μA to 1 mA, and 1 mA–70 mA. It can be used to power external boards that are being measured, with the voltage level configurable between 1.8 V; and 3.6 V with up to 70 mA.

**Figure 1:**
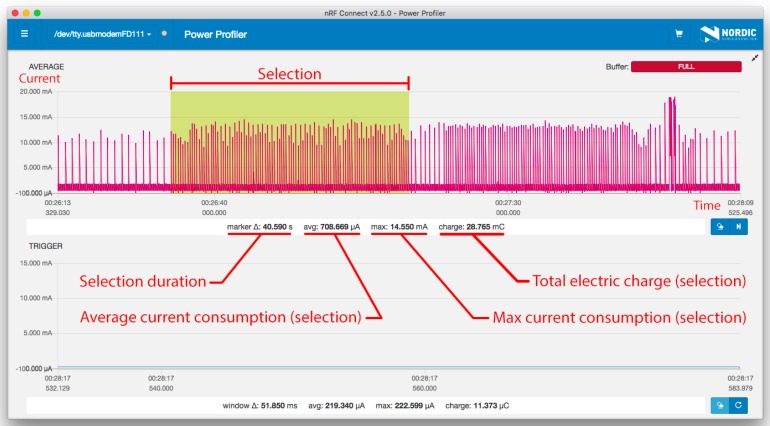
Power Profiler software with test data selected.

Table 1 shows the measurement resolution of *Power Profiler Kit*. It should also be noted that the measurement frequency is 77 kHz, which nets the time resolution of 13 μs, and that the maximum recording/averaging time in the software is 120 s. All tests were conducted in the same environment and under the same conditions. To improve accuracy, the measurements were conducted multiple times and averaged. The measurement data is displayed in two measurement windows: one with a longer acquisition time and one with high-resolution time for high-accuracy plots of triggered events. A subsection of measured data can be selected to average current and energy calculations, as presented in Figure 1.

### 3.3. Hardware Configuration

**Figure 2:**
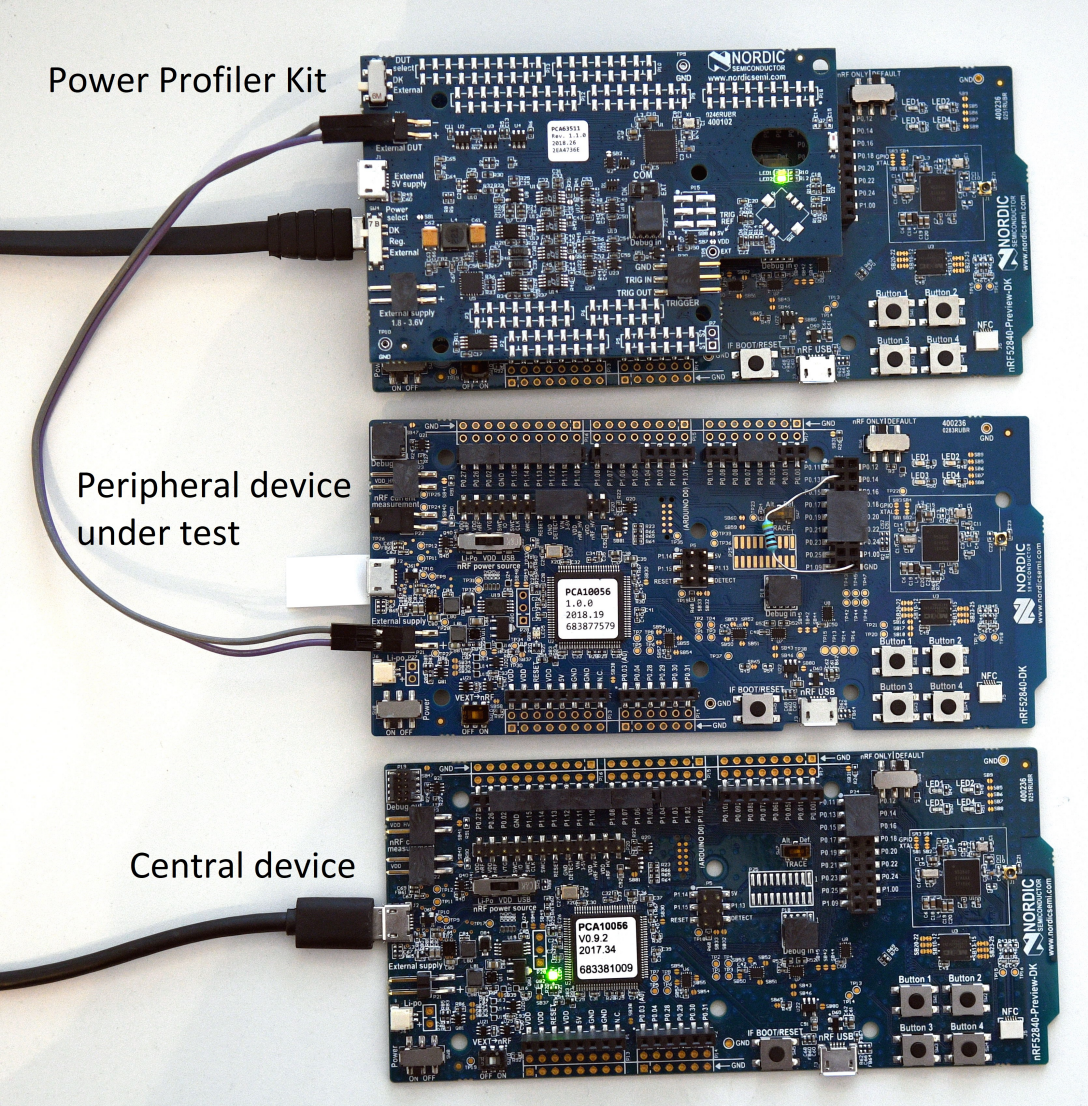
Hardware setup. Top board: Power Profiler Kit on nRF5240 DK; the middle board: peripheral device under test; and the bottom board: central device.

Although *Power Profiler Kit* can run on the peripheral device under test of which the current consumption is measured, we have decided to separate the measurement system (the system on which *Power Profiler Kit* runs) and the peripheral device under test. This was done to avoid any interference that *Power Profiler Kit* might present to the current measurement, as well as the Bluetooth connection. Therefore, we added another nRF52840 DK board for *Power Profiler Kit*. The actual hardware setup used to evaluate the power consumption can be seen in Figure 2. Since *Power Profiler Kit* does not support the internal triggering of the data acquisition system, the measurement interval must be manually selected on the measurement window. To ease the selection of the measurement interval, a programmable load was introduced. The load is a resistor connected between the GPIO pin 13 and ground. The peripheral device under test can be seen on the middle board in Figure 2. While the peripheral device under test is waiting for commands, the GPIO pin 13 is kept high, allowing the current to flow through the resistor and thus increasing the consumption. Once we get the *test start* command, pin 13 is driven low and kept low until the test is finished. The steep transition (drop) in current consumption is used to mark the start and end of the test. An example of how this transition looks in the measurement software can be seen on the left side of Figure 4 where the current consumption falls from ~5 mA to ~0.6 mA and on the right side where the current rises again.

### 3.4. Test Scenarios

In this study, we compare the energy consumption of various BLE versions under the same conditions like radio output power, distance, and orientation of control and peripheral devices. The long range feature introduced in BLE 5 was excluded from the study as earlier BLE versions do not support it.

The data throughput and power consumption of the BLE device are affected by numerous connection parameters. The parameters that most notably affect data throughput are connection interval (CI) and the Attribute Protocol (ATT) Maximum Transmission Unit (ATT_MTU). The ATT_MTU specifies the maximum length of the ATT packet in bytes. The maximum recommended ATT_MTU size for BLE 4.2 and BLE 5 is 247 [31]. This is because the maximum on-air packet size is 255 bytes. If we strip away the L2CAP header (4 bytes) and the link layer header (4 bytes) from an on-air data packet, we are left with an ATT packet of 247 bytes. Therefore, by setting ATT_MTU to 247, we avoid fragmentation of the ATT packet into several on-air data packets [31,34]. In the ATT packet, additional 3 bytes are used for the ATT header and the remaining bytes are used for the data. The number of ATT packets for a given payload is determined by the following equation:

(1) $$packets=\left\lceil \frac{payload}{ATT\_MTU-3}\right\rceil .$$

The older BLE versions (4.0 and 4.1) further limit the ATT_MTU to 23 bytes, which corresponds to a maximum 32-byte on-air packet size and results in a higher payload fragmentation and lower data throughput.

The Connection Interval (CI) is the minimum time between two consecutive Connection Events (CE). During a connection event, BLE devices exchange information like commands, acknowledgements, and payloads. A larger CI results in longer data transmissions and lower data throughput. This is especially evident with read and write transactions, as these transactions take two CEs to transfer each ATT packet. In the first CE, the read/write command is sent to the peripheral device, and in the second CE, the reply/acknowledgement is sent to the central device.

All measurements of read/write transactions with acknowledgement were performed over the complete transaction. The end of the transaction was defined as the point in time when the next transaction of the same type can be conducted. Each data packet transmission lasts two connection intervals: the first one for data packet transmission and the second one for the GATT (Generic Attribute Profile) acknowledgement. Thus, the required time to transmit packets is as follows:

(2) time=2×packets×CI

The CI is, therefore, a very important BLE parameter; however, it mainly affects the BLE performance (especially the throughput) for operations with GATT acknowledgements. Even for these operations, CI and ATT_MTU are equally important. Combining Equations (1) and (2), we can obtain the upper bound of theoretical throughput of BLE operations with GATT acknowledgement as follows:

(3) $$throughput\leq \frac{ATT\_MTU-3}{2\times CI}$$

Note that the domain of CI (7.5 ms–4 s) is larger than the domain of ATT_MTU (23–247 bytes), so it is safe to assume that CI has a bigger impact.

In our experiments, the central device and the peripheral device were preconfigured with the same ATT_MTU parameter. We used the maximum ATT_MTU for each BLE version (23 in the case of BLE 4.0 and BLE 4.1 and 247 in the case of BLE 4.2 and BLE 5). This way, minimal payload fragmentation and shortest transmission time were ensured. From Equations (1) and (2) the number of ATT packets and estimated transmission time for BLE 4.0/4.1 and for BLE 4.2/5 were determined and are presented in Table 2 and Table 3, respectively. Here, it can be seen that, for bigger payloads using long connection intervals, the transmission time exceeds the measuring time limit of *Power Profiler Kit* (120 s). The maximum transmission times that can be measured using *Power Profiler Kit* for given CIs are highlighted in gray.

The BLE connection parameters for test cases were selected using Table 2 and Table 3 and are shown in Table 4. For each BLE version, a payload was selected that enables the measurement of the widest set of CI and, at the same time, provides a sufficient number of ATT packets.

The improvements that BLE 4.1 brought over BLE 4.0 were negligible as far as hardware, throughput, range, and energy consumption are taken into consideration. Because of that, tests for BLE 4.0 and BLE 4.1 were bundled together and done by using the same parameters.

### 3.5. Description of Measurements

All measurements were conducted five times to improve accuracy. The measured values were averaged using the arithmetic mean method. For GATT read and write operations, the measurements were recorded for the time computed using Equation (2) based on the number of transmitted packets. On the other hand, notification transactions and writes without response do not need any GATT level replies and can be chained together. Because of that, the measurements of *notify* and *write without response* operations were stopped when all the data was transferred, even when data transfer was completed in the middle of a CI, as the next transaction can be started immediately after that. For all transactions, we have measured the following quantities: time, average current, energy consumption, and average power. The transaction type, intervals, and measured quantities are summarised in Table 5.

The time taken to transfer the data was reported by the custom firmware running on Bluetooth devices. The time window of the measurement was manually selected using the signal transition within *Power Profiler Kit* as described in Section 3.3. Once the time window was set, the software calculated the total charge consumed and average current in the defined time window. The boards were powered by a power supply with constant voltage of 3.00 V.

We have recorded the duration of the time window *t*, which was marked by the firmware. The average current *I* in the time window has been read from *Power Profiler Kit*. Those two measurements combined with the test scenarios parameters described in Table 4 were used to calculate throughput $throughput=\frac{Payload}{t}$, average power P=VI, and energy consumption E=Pt for each measurement. Measurements were averaged using the mean method, and standard deviation was calculated for all test cases described in Table 4. All results are published on GitHub [35].

**Figure 3:**
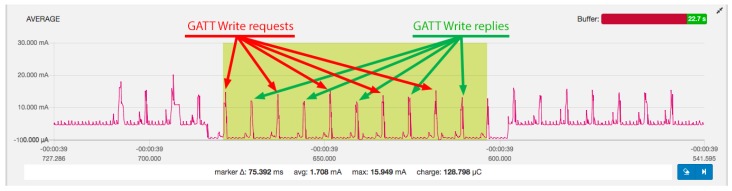
Measurement example: BLE 4.0/4.1 (ATT_MTU: 23), write 100 bytes, CI 7.5 ms. The *Power Profiler Kit* software shows a scale starting at −100μA by default to be able to draw the signal line thickness, even if the signals are always above 0μA.

An example of measurement of current consumption of the BLE 4.0/4.1 device is presented in Figure 3. The GATT requests (red) and replies (green) are annotated in Figure 3. The individual packets are visible, as well as the activity of radio at the start of each connection event. It can also be observed that the GATT Write requests consume a bit more energy than the GATT Write responses. It can also be seen how the peripheral device under test turned off the additional load resistor just before the test started and turned it back on just after the test finished. It should be noted that the link layer reply is sent for each packet to ensure reliability. As predicted by calculations shown in Table 2, five packets were needed to transfer 100 bytes of data, which occupied ten connection events and took 75 ms to complete.

**Figure 4:**
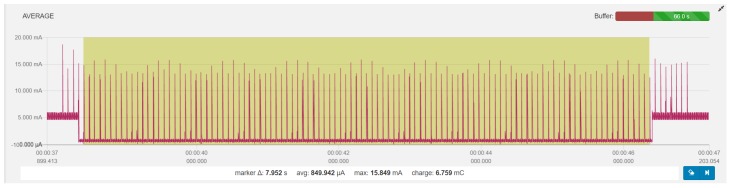
Measurement example: BLE 4.2 (ATT_MTU: 247), read 12.5 KB, CI 75 ms.

An example of power consumption measurements for BLE 4.2, with ATT_MTU set to 247 bytes for the reading of 12.5 KB of payload data using connection interval of 75 ms is depicted in Figure 4. The throughput of the GATT Read operation is bound by the data transfer acknowledgements defined by the BLE protocol. An acknowledgement is generated for each data packet and is transmitted in a separate connection event. The GATT Read operation is started with the GATT Read request in the first CE followed by the CE pairs that correspond to GATT Read reply and GATT reply acknowledgement for each data package. Each data packet in BLE 4.2 can transfer roughly 10x more data than in BLE 4.1 due to the increased ATT_MTU. Consequently, the data throughput of the GATT Read operation in BLE 4.2 is 10x higher compared to BLE 4.1.

**Figure 5:**
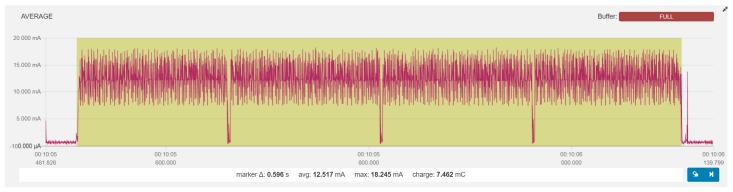
Measurement example: BLE 5 (ATT_MTU: 247), notify 100 KB, CI 150 ms.

Lastly, a measurement example for BLE 5 can be seen in Figure 5. ATT_MTU is set to 247, and 2 Mbps PHY introduced by BLE 5 is used for transferring 100 KB of data. Data was transferred using notifications, with the connection interval set to 150 ms. Because notify operations (opposed to read and write operations) do not need the GATT replies but rely solely on link layer replies, multiple packets can be sent in the single connection event. We can see that the data transmission took just four full connection events.

### 3.6. Firmware Implementation

**Figure 6:**
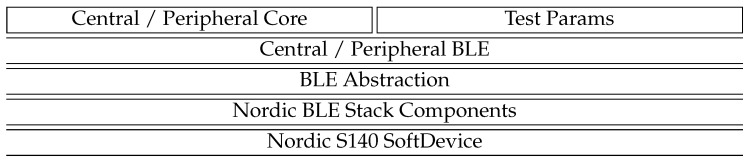
Firmware abstraction stack architecture.

To be able to perform the test described in Table 4, a highly modular firmware was built from scratch. The projects for the central and the peripheral device are separated, but they reuse a lot of lower abstraction layers, which have been built on top of *Nordic BLE Stack Components* to provide an even higher abstraction to the topmost layers. The abstraction stack architecture can be observed in Figure 6.

The layers and their functions are as follows:

- **Central/Peripheral Core** module contains the testing logic. It is implemented using a state machine, which initializes the device on boot and awaits for further instructions.
- **Test Params** module is shared between central and peripheral devices. It is used to load the test parameters for different Bluetooth versions, as well as data building and checking.
- **Central/Peripheral BLE** module maps the internal handles to universally unique identifiers (UUIDs), as well as forwards relevant Bluetooth events to the core layer. On the central device, it is also responsible for scanning, connecting, and service/characteristic discovery procedures.
- **BLE Abstraction** provides a layer of abstraction over the different Nordic BLE Stack Components.
- **Nordic BLE Stack Components** are the host part component abstractions of the BLE Stack, provided by Nordic Semiconductor. These include GATT, GAP, L2CAP, and other BLE layers and modules, as well as a hardware abstraction layer.
- **Nordic S140 SoftDevice** is a pre-compiled binary image. It is functionally verified according to the wireless protocol specification and reveals only its Application Programming Interface (API).

## 4. Results and Discussion

Measurements for various BLE versions and different BLE transactions with and without GATT acknowledgement were performed using a different set of connection parameters, as presented in Table 4. In all experiments, BLE 4.0, BLE 4.1, and BLE 4.2 use the same 1 Mb/s PHY, while BLE 5 uses 2 Mb/s PHY. First, we present the effect of CI on throughput for different BLE versions. Then, we present the effect of throughput on power and energy consumption.

### 4.1. Throughput

The primary means of changing throughput in BLE are ATT_MTU, CI, and PHY modulation speed. The Bluetooth version limits the ATT_MTU values as well as PHY modulation. To confine the number of experiments, we bound the ATT_MTU parameter to its highest value for the BLE version and chose the PHY modulation with the highest rate. This way, the highest throughput for a given BLE version is achieved. The only unspecified BLE parameter that affects the data throughput is CI.

**Figure 7:**
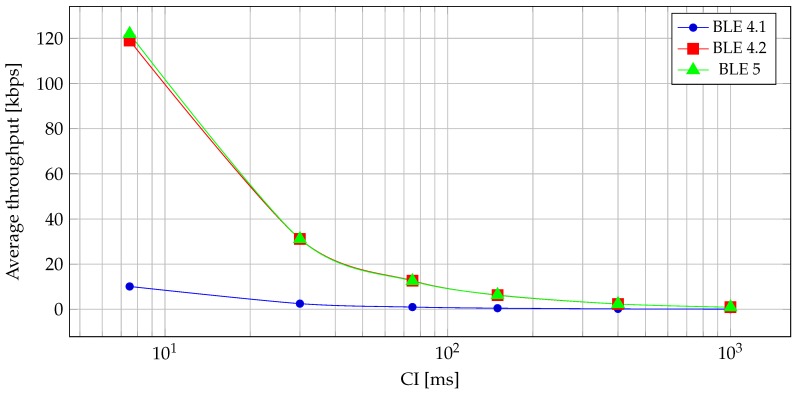
Average read throughput of BLE versions at different CIs.

The throughput of read/write transactions is limited by the fact that a GATT acknowledgement is required and it is sent in the following CI. The relationship between data throughput and CI is presented in Figure 7. We can see that there is a clear correlation between CI and throughput. Figure 7 was generated for the read operation; however, the write operation exhibits the same property.

**Figure 8:**
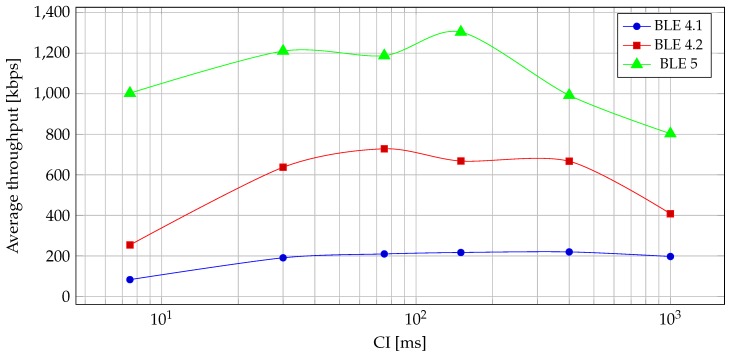
Average notification throughput of BLE versions at different CIs.

Notifications, on the other hand, are not limited by the GATT acknowledgements, as they rely solely on link layer acknowledgements, which are sent in the same CE. This means that, in theory, if we can transmit all the data in a single CE, the highest throughput will be achieved. In practice, this is not true, as it can be seen in Figure 8. For notifications, a CI that is too short will decrease the throughput because of extra overhead needed to open and close the connection event too often. On the other side, a CI that is too long will, in practice, decrease throughput because of interference and packet failures. The reason for decreased throughput, in that case, is that a CE is closed when a packet failure happens and that the packet is retried on the next CE, meaning that the rest of the closed CE is unused; thus, the average time of transmission increases with the increase of CI. A decrease in throughput, after CI exceeds some threshold, can be observed in Figure 8.

The theoretical maximum data throughput of different BLE versions was studied in Reference [29]. The results are summarized in Table 6, where the modulation rate and max throughput are given. Comparing these results with our measurements, we can see that the peak performance of each BLE version is close to the theoretical maximum.

### 4.2. Power End Energy Consumption For Read and Write Transactions

Read and write operations require a GATT acknowledgement that is generated in a separate CE. During a single CE, they both transmit either a single packet or a GATT acknowledgement. Therefore, observations made on the read operation can be generalized to the write operation and vice versa. For this reason, we have included a single graph that illustrates average power consumption as a function of data throughput.

**Figure 9:**
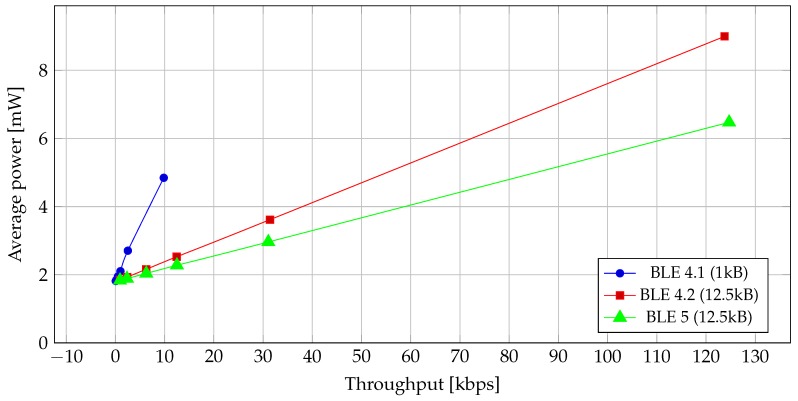
Average power vs. data throughput for read/write operation. Note: Data size and transmit time are different for BLE 4.1 and BLE 4.2/BLE 5.

The relationship between power consumption and data throughput for different BLE versions is given in Figure 9, where we can see that power efficiency improved with each newer BLE version. It is interesting to see that, at the same throughput, BLE 5 uses less power than BLE 4.2 even though it uses 2 Mb/s PHY modulations which draws more power. That is because the transmission time of a single packet has almost halved in comparison to BLE 4.2, as seen in Table 6. The shorter transmission time of a packet means that the device can turn off the radio and go into power-saving mode sooner, which in turn reduces the power consumption. Even with fragmented transmissions, which are present in read/write operations, this effect prevails over the increase in momentary power consumption, thus reducing overall power consumption.

**Figure 10:**
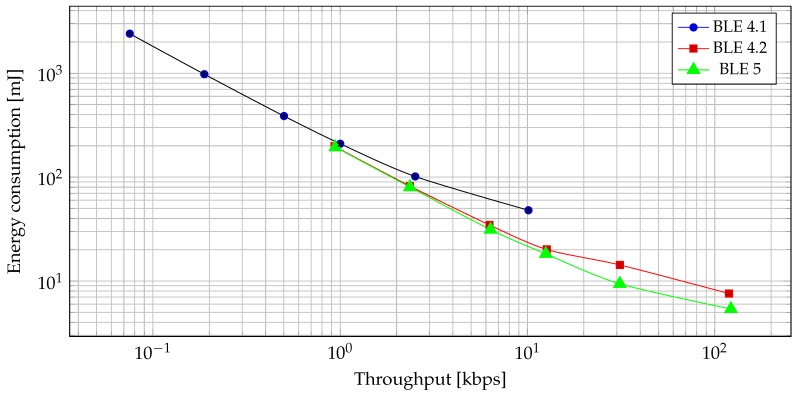
Energy consumption vs. throughput for read/write operation.

Another interesting thing to observe is just how much BLE has evolved with 4.2 and 5, both in throughput and power consumption domains. This can be seen in Figure 10, where we can observe that the energy needed to transmit a certain amount of data lowers with each Bluetooth version.

### 4.3. Power and Energy Consumption for *Notify* and *Write without Response*

*Notify* and *write without response* are very similar operations. The main difference is the direction of data, which in our case does not play a significant role. They are the fastest operations that are supported by the majority of BLE devices because they are not limited by waiting for higher layer acknowledgements. They still do need to respect the connection event start/endpoints as well as the fact that if a packet transmission fails in the current CE, the CE will be prematurely ended and that the transmission of a failed packet will be retried in the next CE, with the queued packets being moved to the next CE as well.

**Figure 11:**
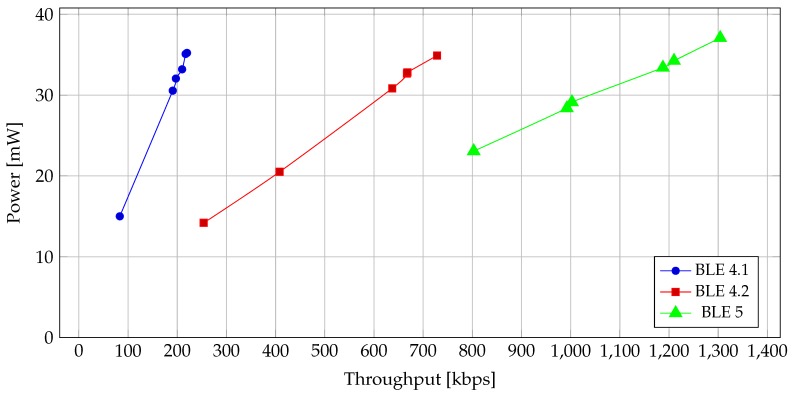
Power vs. throughput for different BLE versions for *notify* and *write without response*.

**Figure 12:**
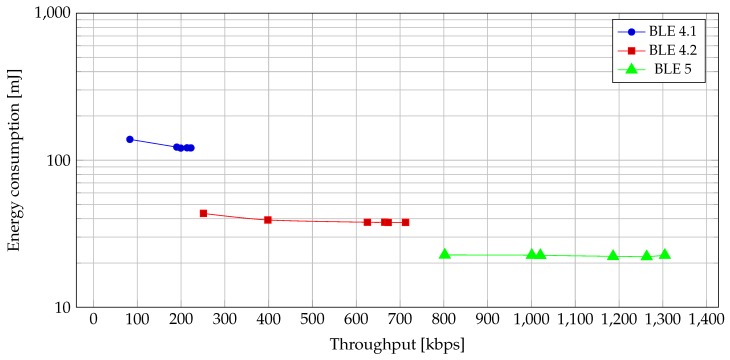
Energy vs. throughput for different BLE versions for *notify* and *write without response*.

With *notify* and *write without response*, the differences between Bluetooth versions become much more noticeable, as seen in Figure 11 and Figure 12. Because in these two operations the CEs are filled with packets, the differences in throughput as well as power consumption are more apparent.

In Figure 11, we can compare the power consumption vs. throughput for different BLE versions. We can see a leap of performance between BLE 4.1 and BLE 4.2. This is due to the increase of the maximal ATT_MTU in BLE 4.2, which increases the ratio of user data over packet size and enables BLE 4.2 to have a higher throughput. Comparing BLE 4.2 and BLE 5, we can see that they use different PHY layers. Here, we can see that the 2 Mb/s PHY used in BLE 5 consumes more power. On the other hand, it also nets significantly higher throughput (roughly 1.7x increase).

From Figure 12, we can see that the energy consumption for transmission of a single payload has been lowered substantially with each BLE version. The first significant drop in energy consumption is between BLE 4.1 and BLE 4.2 versions and can be attributed solely to the increase of ATT_MTU. This change increases the size of packets and decreases the number of packets to be sent for a given amount of data. Since each packet has some fixed overhead, the decrease in the number of packets corresponds to the decrease of total data transmitted. This reduces total energy consumption as well as transmission time for the payload. BLE 5 lowers the energy consumption for transmission of the same amount of data even further. As for BLE 4.2, this can be attributed to shorter transmission time. The latter is achieved by using a more capable PHY modulation with a higher rate. Even though the power consumption of the more capable PHY is higher (Figure 11), the reduction in the overall time required for transmission leads to lower energy consumption (Figure 12).

From Figure 12, we can deduce another interesting and useful property: individual BLE versions use approximately the same amount of energy for transmission of a given payload regardless of throughput or selected CI. This information can be beneficial, as it enables us to tune our application to suit our needs in regards to throughput and the latency of read/write commands without worrying about energy consumption.

## 5. Conclusions and Future Work

The recently introduced BLE 5 promises twice the speed, four times the range, and eight times the broadcast capacity, all of which is supposed to leave power consumption unaffected [23]. Because we did not find any research that compares the effect of different connection parameters and BLE versions on energy consumption regarding throughput, we decided to perform such an investigation. We have investigated the effect of the CI parameter on the throughput for the read/write and *notify/write without response* transactions. We have found that lowering CI will increase throughput for reads and writes, but that is not the case for notifications and writes without response. We have also performed the tests needed for comparing power efficiency regarding throughput for various BLE versions and transactions. We grouped the comparisons by the type of transaction. That is because the read/write transactions are conceptually similar operations, with the difference being the data flow direction, and the same goes for the *notify/write without response* transactions.

It has been shown that energy consumption reduces with each newer BLE version, even though BLE 5, configured to use the 2 Mb/s PHY, consumes more power than BLE 4 when radios are operating (i.e., higher transmission rates require more power). On the other hand, BLE 5 transmits the same amount of data in a shorter time. Since the power increase is smaller than transmission time decrease, the overall energy consumption for a given data volume is lower. Furthermore, for each BLE version, it was demonstrated that energy consumption is affected mostly by the data volume. Similarly, maximum data throughput rises with each newer BLE version. However, the data throughput varies also with the selected transaction: the throughput of transactions with the GATT acknowledgements mainly depends on CI, while the data throughput of transactions without the GATT acknowledgements does not change significantly over the range of CI.

The findings in this work can be beneficial, as they enable us to tune our application to suit our needs in regards to throughput without worrying about energy consumption when large amounts of data need to be transmitted. As far as we know, this work is one of the first to provide the measurements of power consumption with different CIs and data throughput for various BLE versions. We strongly believe that this information is required to provide a full design guideline for implementing an optimal BLE IoT application.

In the experiments performed in this study, we focused on the comparison of different BLE versions in terms of energy consumption and throughput. To do this, we used the same placement of devices, the same radio power, and other BLE parameters except for BLE parameters that characterize the BLE version. Our goal was to perform a comparative study in an ideal (or near ideal) situation, where connections are fault-free and result in the highest possible throughput. However, obstacles and interference can significantly affect the BLE transactions and we intend to include obstacles and interference in future work.

## Figures and Tables

**Table 1:** *Power Profiler Kit* measurement resolution.

Range	Resolution
1–70 μA	0.2 μA
70 μA–1 mA	3 μA
1–70 mA	50 μA

**Table 2:** Packets and time needed to transfer different payloads in relation to connection interval (CI) for BLE 4.0/4.1 with ATT_MTU set to 23, using read/write operation. The maximum transmission times that can be measured using *Power Profiler Kit* for given CIs are highlighted in gray.

*Payload*	*Packets*	Time (s) @ CI				
		@ 7.5 ms	@ 50 ms	@ 400 ms	@ 1000 ms	@ 4000 ms
20 B	1	0.015	0.1	0.8	2	8
100 B	5	0.075	0.5	4	10	40
400 B	20	0.3	2	16	40	160
1 KB	50	0.75	5	40	100	400
10 KB	500	7.5	50	400	1000	4000
100 KB	5000	75	500	4000	10000	40000

**Table 3:** Packets and time needed to transfer different payloads in relation to CI for BLE 4.2/5 with ATT_MTU set to 247, using read/write operation. The maximum transmission times that can be measured using *Power Profiler Kit* for given CIs are highlighted in gray.

*Payload*	*Packets*	Time (s) @ CI				
		@ 7.5 ms	@ 50 ms	@ 400 ms	@ 1000 ms	@ 4000 ms
20 B	1	0.015	0.1	0.8	2	8
100 B	1	0.015	0.1	0.8	2	8
400 B	2	0.03	0.2	1.6	4	16
1 KB	5	0.075	0.5	4	10	40
10 KB	41	0.615	4.1	32.8	82	328
12.5 KB	52	0.78	5.2	41.6	104	416
100 KB	410	6.15	41	328	820	3280

**Table 4:** Test cases with their parameters.

BLE Version	Constant Parameters	CI (ms)	Transaction	Payload
4.0/4.1	ATT_MTU: 23 TX power: 8 dBm	7.5, 30, 75, 150, 400, 1000	Read, Write	1 KB
	PHY: 1 Mbps Distance: 2 cm		Notify Write w/o rsp	100 KB
4.2	ATT_MTU: 247 TX power: 8 dBm	7.5, 30, 75, 150, 400, 1000	Read, Write	12.5 KB
	PHY: 1 Mbps Distance: 2 cm		Notify Write w/o rsp	100 KB
v5	ATT_MTU: 247 TX power: 8 dBm	7.5, 30, 75, 150, 400, 1000	Read, Write	12.5 KB
	PHY: 2 Mbps Distance: 2 cm		Notify Write w/o rsp	100 KB

**Table 5:** Parameters of measurements for test cases described in Table 4.

Transaction	Measurement Interval	Measurements
Read Write	From the first GATT request to the end of CI of the last GATT reply	Time Average current Energy consumption Power consumption
Notify Write w/o rsp	From the start of first GATT request to the end of last GATT request	

**Table 6:** Maximum theoretical throughput for different BLE versions [29].

BLE Version	Modulation Rate	Max Throughput
4.0/4.1	1 Mb/s	0.305 Mb/s
4.2	1 Mb/s	0.803 Mb/s
5	2 Mb/s	1.4 Mb/s

## Abbreviations

The following abbreviations are used in this manuscript:

BLE	Bluetooth Low Energy
SoC	System on Chip
IoT	Internet of Things
CI	Connection Interval
CE	Connection Event
DUT	Device Under Test
ATT	Attribute Protocol
GATT	Generic Attribute Profile
